# The Denuding of Teeth

**Published:** 1882-03

**Authors:** Geo. W. M'Elhaney

**Affiliations:** West Point, Ga.


### ARTICLE IV.

### *The Denuding of Teeth.*

BY GEO. W. M'ELHANEY, D. D. S., WEST POINT, GA.

[Read before the Georgia State Dental Association.]

If I were competent I would like to present to this Association an article on a subject that is rarely written upon, and I do not call to mind now of ever hearing it discussed in either our National or State societies; and feeling myself unqualified to do the subject justice, I shall partially fulfill a duty that I owe to this Association as a member appointed on one of the committees to write some article on Dentistry, by simply calling your attention to it, hoping that by some discussion we all will be profited. It is "the denuding of the teeth."

There are few of us but who have been puzzled and perplexed when this curious defect in nature (for I know of no better name to call it) comes before us. And how shall I write about a disease of which our text books and great lights in dental literature have said so little? That great writer, who, for simplicity and preciseness, has, to my feeble conception, written the greatest work on Dentistry up to the present day, Chapin A. Harris, describes it so plainly that a blind man might be able to diagnose it, and still gives us nothing that we can grasp hold upon as to its cause. After telling us what Hunter, Bell, and Fox's theories are, and that they are not correct, he gives us nothing better for himself as to its cause; but as to its treatment, I think he is correct.

I have been led to think no little upon this disease, more, I suppose, on account of some perplexing cases that have come before me than anything else, where all theories as to its causes are at fault, according to my judgment, and which lead me to think that perhaps there is a cause, and it is our duty to find it out, not that I can present even a new theory. It is said that Mr. Hunter is the first writer who notices this curious affection of the teeth. He calls it "decay by denudation," on account of its being confined to particular teeth; and it is a disease inherent in the tooth itself and not dependent on circumstances.

Mr. Fox, another great writer, frankly says that he does not know any cause for it, but thinks it dependent on some solvent quality of the saliva.

Mr. Harris says (and I think I might say all of us, years after these views were written, will fully agree with him) that this conjecture will not do, for every part of the tooth would be alike subject to these attacks, whereas we know it is principally confined to the central incisors and laterals, and sometimes on around even to and attacking the second molars.

Some writers think it is caused by friction of the lips. Some, again, think it is caused by the use of the toothbrush. Mr. Bell says that Mr. Hunter has confounded this affection with another, and attempts to explain it; and then Mr. Harris says that he, in attempt to correct Mr. Hunter, gets into error himself. But, still, Mr. Harris shows us nothing definite, and although it is many long years since these views were written, we are still groping in the dark as to its cause. Hence, I say, here is a field for exploration.

I have a patient (the daughter of an intelligent physician who takes great interest in the health regimen of his children,) a bright little girl nine years old, and fully developed, physically and mentally, for age; teeth of a dense structure, prettily shaped, whose central incisors have been attacked with this disease, a well developed groove horizontally across the middle of the tooth. Now the laterals are about two-thirds developed, and right where they touch the centrals the disease ceases, but dives down into the lateral edge of the incisors and exposes the dentine. Further up the groove, toward the centre of the tooth, the denudation (as I will express it) is all covered with a thin, knotty appearance of enamel. Now these teeth are not fully developed, and the question which presents itself to me is, how shall I treat them? Well, I have separated the laterals on both sides, and at the touching edges of these centrals, find them decayed (having the yellowish color that Harris describes,) and have filled them with gold; but have not followed on this groove, trusting that, as the child grows older, nature might do something, and I believe by separating and polishing these laterals I shall arrest the denudation from any other ravages on the other teeth.

I have talked freely with the father of this child, and nothing was ever on his (or his parents') teeth; and her mother was a patient of mine long before her marriage, and nothing of the kind ever affected her teeth. Now arises the question, what caused this disease on this child's teeth? None of the above mentioned theories can be applicable to her case. Now, I want to know if it could not be possible that when this little girl was, say, from two to five years old, she had a fall, striking something very hard with her two front temporary teeth, or all of them, causing such a force as to drive up these temporary teeth against the sacks containing the permanent ones lying so snugly above, awaiting the call of nature to arouse them to do her work at their appointed time? I say, could she not have received a fracture, and thence inflammation, although it would hardly be noticeable (and who notices a child at this age so closely?) and some action of saliva so affected these as to destroy the enamel and cause this? Who knows the many, many hard licks that children get through the carelessness of their nurses? You look at a cut of these teeth and you will see even at the age of three years quite a mixture of these roots of the temporary teeth up among the permanent ones. I believe there is something in this idea, and if I can set others to thinking on the subject I will feel that I have accomplished some good. I would like for us as a society to strike out on new lines, and quit our old *stereotyped* subjects, giving the young men who are coming into the profession something to think about, so that it will be of advantage to the coming dentist as well as ameliorating the condition of humanity.—*Dent. Luminary.*

